# Characterisation of Human Embryonic Stem Cells Conditioning Media by ^1^H-Nuclear Magnetic Resonance Spectroscopy

**DOI:** 10.1371/journal.pone.0016732

**Published:** 2011-02-09

**Authors:** David A. MacIntyre, Darío Melguizo Sanchís, Beatriz Jiménez, Rubén Moreno, Miodrag Stojkovic, Antonio Pineda-Lucena

**Affiliations:** 1 Structural Biochemistry Laboratory, Centro de Investigación Príncipe Felipe, Valencia, Spain; 2 Cellular Reprogramming Laboratory, Centro de Investigación Príncipe Felipe, Valencia, Spain; University of Bristol, United Kingdom

## Abstract

**Background:**

Cell culture media conditioned by human foreskin fibroblasts (HFFs) provide a complex supplement of protein and metabolic factors that support *in vitro* proliferation of human embryonic stem cells (hESCs). However, the conditioning process is variable with different media batches often exhibiting differing capacities to maintain hESCs in culture. While recent studies have examined the protein complement of conditioned culture media, detailed information regarding the metabolic component of this media is lacking.

**Methodology/Principal Findings:**

Using a ^1^H-Nuclear Magnetic Resonance (^1^H-NMR) metabonomics approach, 32 metabolites and small compounds were identified and quantified in media conditioned by passage 11 HFFs (CMp11). A number of metabolites were secreted by HFFs with significantly higher concentration of lactate, alanine, and formate detected in CMp11 compared to non-conditioned media. In contrast, levels of tryptophan, folate and niacinamide were depleted in CMp11 indicating the utilisation of these metabolites by HFFs. Multivariate statistical analysis of the ^1^H-NMR data revealed marked age-related differences in the metabolic profile of CMp11 collected from HFFs every 24 h over 72 h. Additionally, the metabolic profile of CMp11 was altered following freezing at −20°C for 2 weeks. CM derived from passage 18 HFFs (CMp18) was found to be ineffective at supporting hESCs in an undifferentiated state beyond 5 days culture. Multivariate statistical comparison of CMp11 and CMp18 metabolic profiles enabled rapid and clear discrimination between the two media with CMp18 containing lower concentrations of lactate and alanine as well as higher concentrations of glucose and glutamine.

**Conclusions/Significance:**

^1^H-NMR-based metabonomics offers a rapid and accurate method of characterising hESC conditioning media and is a valuable tool for monitoring, controlling and optimising hESC culture media preparation.

## Introduction

Human embryonic stem cells (hESCs) harbor the capacity to differentiate into all primary human cell types [1], [2], [3] and can be cultivated *in vitro* indefinitely under specified culture conditions [4]. This endowers them with the potential to provide an ongoing source of cells for the study of early human development, disease states as well as for applications in drug screening and regenerative medicine [5], [6], [7]. Undifferentiated hESCs are difficult to expand in culture without the presence of different types of fibroblasts [3] or high concentration of different growth factors [8]. However, concerns regarding cross-species virus transfer and subsequent compatibility for therapeutic applications [9], [10], [11], [12] have led to hESCs being typically maintained on human fibroblast feeders or on naturally derived matrices supplemented with media conditioned by fibroblasts [13], [14], [15]. Intuitively, fibroblasts likely secrete a plethora of factors critical for the proliferation and maintenance of hESCs *in vitro*.

In practice, the preparation of HFF conditioned media (CM) is variable and batches of media often exhibit differing capacity to maintain hESC proliferation *in vitro*. Currently this information cannot be garnered until days after initiating the culturing procedure. Consequently, a number of recent studies have attempted to identify protein factors secreted by mouse and human feeder fibroblasts produced during the conditioning process [16], [17], [18], [19] with the ultimate goal of creating a fully synthetic media that can provide robust and consistent maintenance of hESCs. For example, proteomic analysis of CM derived from human neonatal fibroblasts revealed a complex mixture of growth factors, extracellular matrix proteins and differentiation factors potentially involved in the derivation of hESCs [18]. While such investigations have shed light on important protein components of CM, a paucity of information exists regarding the metabolite component of CM. This information is critical for establishing truly chemically defined, robust and consistent hESCs supporting media.

The process of analysing and profiling metabolite concentrations in a biological system is often termed “metabonomics” [20]. This entails the acquisition of the metabolic profile through a multiparametric technological platform such as mass spectrometry [21], [22] or ^1^H-nuclear magnetic resonance (^1^H-NMR) spectroscopy [23], [24], [25], [26] combined with multivariate statistical analysis for the purposes of pattern recognition and pathway analysis [20], [27]. ^1^H-NMR spectroscopy is a particularly advantageous technique in that it is inherently quantitative, reproducible, minimal sample preparation is required and samples can be recovered following analysis. Characterisation of cell culture media by ^1^H-NMR has facilitated the detailed scrutiny of metabolites involved in a variety of biochemical pathways [28], [29], [30] and most recently, has been used to examine media conditions associated with human hepatoma cell growth *in vitro* [31] and for potential tissue engineering applications [32].

In this study, we describe the first application of ^1^H-NMR-based metabonomics for the characterisation of the metabolite component of CM derived from human fibroblasts. Specifically, we aimed to characterise the metabolite footprint (i.e. those metabolites secreted and/or utilised by foreskin cells- of human foreskin cells during the conditioning process) of CM and through the use of multivariate statistical methods, to model the conditioning process against time. This approach also enabled us to examine changes in CM induced by freeze storage and to identify functionally critical metabolic characteristics of supportive CM compared with non-support CM. Our results indicate ^1^H-NMR-based metabonomics can be used for characterising metabolic constituents of CM, which facilitates the preparation and recognition of functionally supportive CM. The method may also be useful for formulating an optimised and chemically defined hESC and induced pluripotency stem cell (iPSC) culture media suitable for therapeutic application or drug development/screening.

## Results

**Figure 1:**
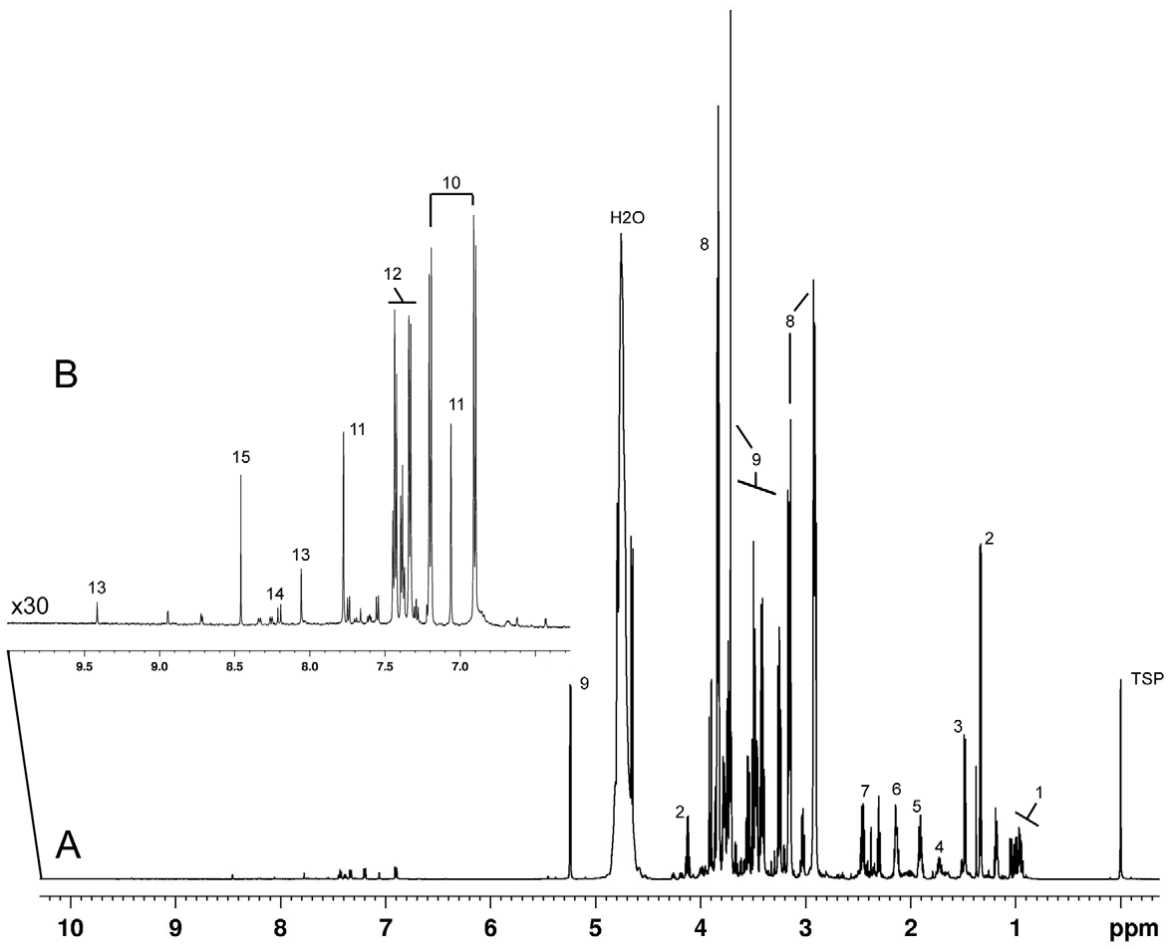
Representative 600 MHz 1D-CPMG^ 1^H NMR spectra of TeSR-1 collected after 24 h conditioning by human fibroblasts. (A) Full spectra (0.00–10.00 ppm) and (B) magnification (×30) of aromatic region (6.00–9.00 ppm). Using the NMR Metabolic Profiling Database (Bruker) along with in-house standards, 42 metabolites could be assigned to the spectra. Numbers refer to the more abundant metabolites including; 1) branched amino acids including valine, isoleucine and leucine, 2) lactate, 3) alanine, 4) lysine, 5) acetate, 6) methionine, 7) glutamine, 8) HEPES, 9) glucose, 10) tyrosine, 11) histidine, 12) phenylalanine, 13) thiamine, 14) niacinamide and 15) formate. Spectra were internally calibrated to TSP at chemical shift δ = 0.00 ppm. For further details of metabolites assignments see Table 1.

**Figure 2:**
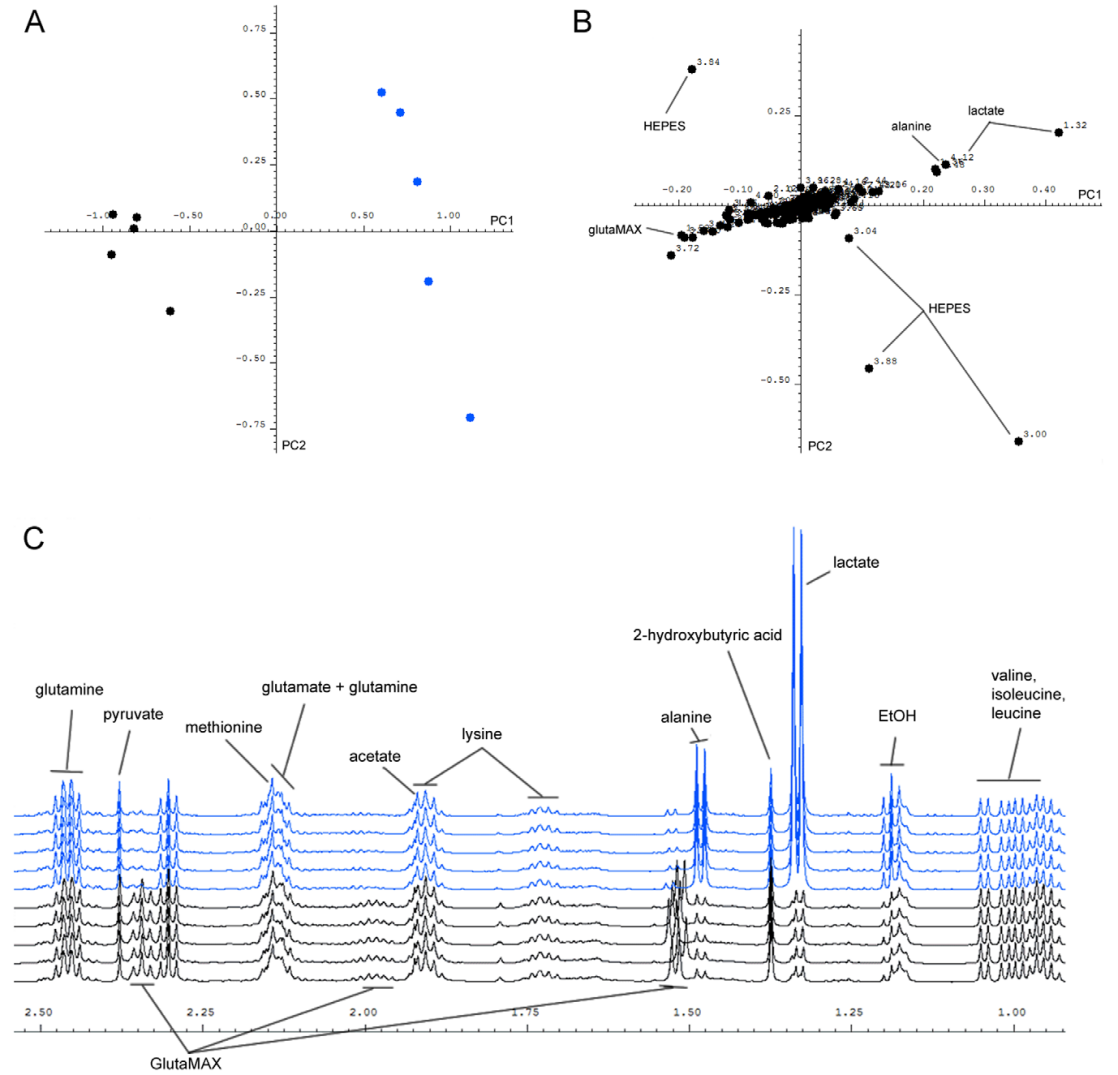
Comparison of metabolic profiles from unconditioned TeSR-1 media (black) and CMp11 (blue). Initial analysis of the metabolic “fingerprints” of unconditioned TeSR-1 media (UM) and conditioned media (CMp11) was performed using unsupervised PCA. (**A**) Examination of the PCA score plot revealed marked differences between 5 batches of UM and CMp11. (**B**) Analysis of the corresponding PCA loadings plot enabled rapid recognition of those regions of the ^1^H-NMR spectra responsible for this separation. The metabolites were then identified and their concentrations calculated (see Table 1). (**C**) A magnified region of the ^1^H-NMR spectra showing characteristically higher concentrations of lactate and alanine as well as decreased concentrations of the commercial L-glutamine source, GlutaMAX^TM^ present in CMp11.

A representative 1D-CPMG ^1^H-NMR profile of TeSR-1 media collected following 24 h of conditioning by HFFs is presented in Figure 1. Around 250 spectral features corresponding to proton signals derived from a range of small compounds (collectively referred to from here on as metabolites) could be observed. The most abundant metabolites in the TeSR-1 media conditioned by passage 11 HFFs (CMp11) are labelled and include energy substrates, amino acids and vitamins. It should be noted that this spectra collectively represents all metabolites present and detectable by ^1^H-NMR in TeSR-1 media prior to conditioning (see Figure 2C for visual comparison of the TeSR-1 media before and after conditioning).

To facilitate the rapid examination of global metabolic changes in the media following conditioning, ^1^H-NMR spectral profiles from batches of unconditioned (UM) and conditioned CMp11 samples were analysed by PCA. The PCA score plot showed clear separation between UM and CMp11 samples with the former clustering in the left quadrant and latter in the right quadrant indicating marked differences in the underlying metabolic profiles (Figure 2A). Analysis of the corresponding PCA loadings plot revealed those metabolites primarily responsible for this separation (Figure 2B). As NMR spectroscopy is inherently quantitative, changes in peak signal intensity are directly correlated to concentration. Thus, interpretation of PCA loadings indicates both directional changes in peak intensities and concentrations of metabolites between samples. Characteristically, CMp11 contained higher concentrations of lactate and alanine whilst UM samples contained higher concentrations of GlutaMAX^TM^. Consistent changes in the chemical shift of ^1^H signals originating from HEPES were also observed in CMp11 indicating changing pH during the conditioning process. These aforementioned changes were also evident following direct visual inspection of the CPMG ^1^H-NMR spectra (Figure 2C).

To further characterise metabolic changes in TeSR-1 media induced by conditioning, quantification of metabolites was performed using NOESY ^1^H-NMR spectra acquired from the same media samples. Accuracy of this method was successfully confirmed by comparing the calculated concentrations of 20 metabolites in TeSR-1 media with those values acquired experimentally (see Table S1). Table 1 describes quantification of 32 metabolites detectable in media samples as well as the chemical shifts and assignment of signals used for the calculation of concentrations. Changes induced by HFF conditioning on the TeSR-1 media included the production and secretion of metabolites (observed as significantly higher concentrations) as well as the sequestering and utilisation of others (detected as significantly lower concentrations). For example, the concentration of lactate, alanine, formate, pyruvate, and pyroglutamic acid were all determined to be significantly higher following 24 h conditioning indicating their production and secretion by HFFs. Conversely, the concentration of GlutaMAX^TM^, tryptophan, niacinamide and pyridoxine were significantly lower in CM indicating utilisation by HFFs. Interestingly, 2-hydroxyisobutyric acid and 2-hydroxyisovaleric acid, neither of which are included in published recipes of TeSR-1 media, were detected by ^1^H-NMR in UM.

**Table 1:** Concentration (mM ± SD) of metabolites detected in TeSR-1 media before and after conditioning with HFFs for 24 h (CM) using ^1^H NMR.

Metabolites	TeSR-1 [mM]	CM [mM]	P Value	[Change]	Chemical shift (ppm)	Multiplicity	Assignment
**Amino Acids**							
alanine*	0.34±0.02	1.50±0.03	<0.001	↑	1.47	d	CH_3_
arginine*	1.5±0.2	1.2±0.2	0.051	↓	1.68–1.75	m	γ-CΗ_2_
glutamate	0.56±0.06	0.71±0.02	0.012	↑	2.08	m	half β-CH_2_
GlutaMAX™	1.21±0.11	0.35±0.02	<0.001	↓	1.52	d	CH_3_
glutamine	2.1±0.2	2.80±0.07	0.004	↑	2.45	m	half γ-CH_2_
glycine	0.47±0.07	0.58±0.02	0.032	↑	3.56	s	CH_2_
histidine	0.12±0.01	0.127±0.002	0.152	↔	7.07	s	H4
isoleucine	0.36±0.04	0.32±0.01	0.167	↔	1.00	d	β-CH_3_
leucine	0.37±0.04	0.33±0.01	0.119	↔	0.97	d+d	−δCΗ_3_
lysine*	1.1±0.1	1.13±0.04	0.730	↔	1.75	m	−δCΗ_2_
methionine	0.13±0.02	0.12±0.01	0.339	↔	2.65	t	−γCΗ_2_
phenylalanine	0.20±0.02	0.205±0.004	0.420	↔	7.39	m	H2,3,4,5+6
serine*	0.80±0.09	0.82±0.07	0.865	↔	3.98	m	−βCΗ_2_
threonine**	0.16±0.06	0.29±0.05	0.023	↑	4.26	m	−βCΗ
tryptophan	0.036±0.002	0.029±0.001	0.002	↓	7.75	d	CH
tyrosine	0.20±0.02	0.209±0.004	0.305	↔	7.20	d	CH
valine	0.36±0.03	0.35±0.01	0.723	↔	1.03	d	CH_3_
**Vitamins**							
choline	0.065±0.006	0.062±0.002	0.384	↔	3.21	s	N(CH_3_)_3_
folate	<0.001	-	0.050	↓	6.42	d	CH
myo-inositol	0.035±0.007	0.040±0.005	0.214	↔	4.07	s	H5
niacinamide	0.010±0.001	0.0081±0.0005	0.024	↓	8.94	s	NCH
pantothenic acid	0.014±0.002	0.024±0.001	0.001	↑	0.89	s	CH_3_
pyridoxine	0.013±0.002	0.010±0.001	0.017	↓	7.66	s	H6
thiamine	0.012±0.002	0.0138±0.0009	0.125	↔	9.42	s	CH
**Other**							
2-hydroxyisobutyric acid	0.32±0.03	0.29±0.01	0.155	↔	1.37	s	CH_3_
2-hydroxyisovaleric acid	0.007±0.002	0.007±0.001	0.849	↔	0.84	d	CH_3_
formate	-	0.079±0.001	<0.001	↑	8.46	s	CH
glucose total	16±2	14.6±0.5	0.196	↓	5.24 + 3.9	d + dd	H1 + half CH2-C6
HEPES	13.3±1.3	14.4±0.4	0.166	↔	3.12–3.18	m	CH_2_(SO_3_)
lactate	-	4.46±0.09	<0.001	↑	4.12	q	CH
pyroglutamic acid	-	0.20±0.01	<0.001	↑	2.40	m	CH_2_
pyruvate	0.27±0.03	0.31±0.01	0.049	↑	2.38	s	CH_3_

1D-NOESY spectra (one run per sample) were acquired using a Bruker Avance II 600 spectrometer equipped with a TCI cryoprobe. Concentration values were determined by referencing to the internal standard, TSP. The concentration of some metabolites was overestimated due to co-resonant peaks (*) or baseline distortion due to the close proximity of the water signal (**). Changes in concentration following conditioning are indicated by directional arrows (↑↓).

Abbreviations: s-singlet; d-doublet; dd-double doublet, t-triplet; q-quartet; m-multiplet. Chemical shifts referenced to TSP at 0.00 ppm. Values reported as mean values (n = 5) ± standard deviation (SD).

**Figure 3:**
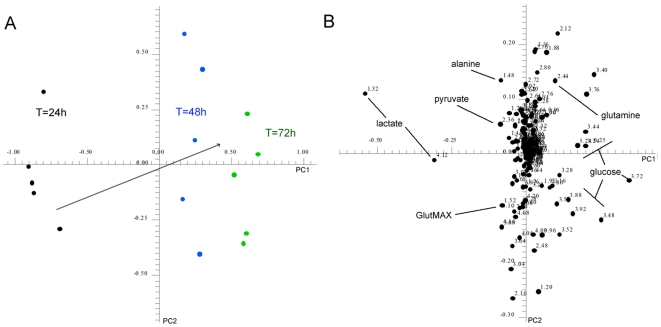
Changes in the metabolic profile of CMp11 during a time course of 3 days. Samples of CMp11 were collected at 24 h intervals for 3 days. Following each collection, culture media was replaced with fresh TeSR-1 media (**A**) PCA analysis revealed a clear time related differences in the metabolic component of CMp11. (**B**) Analysis of corresponding loadings plots showed decreased formation of lactate and pyruvate, decreased utilisation of glucose, and increased concentrations of acetate and EtOH were characteristic of samples collected after 72 h.

As conditioned media is often collected and pooled over a number of days, we investigated changes in the metabolic profile of CMp11 collected over a time course of 3 days. A rapid analysis of metabolic changes of TeSR-1 media samples collected every 24 h for 3 days was achieved through the application of PCA to the NMR-acquired data. As seen in the PCA score plot (Figure 3A), distinct differences in the metabolic profiles of media samples collected over time were observable. Reference to the corresponding loadings plots showed that media samples collected at 24 h contain comparatively higher concentrations of glycolysis endpoints such as lactate, alanine and pyruvate than 48 h and 72 h time points. Consistent with this, metabolic profiles of the 48 h and 72 h samples consisted of higher concentrations of glucose and glutamine.

**Figure 4:**
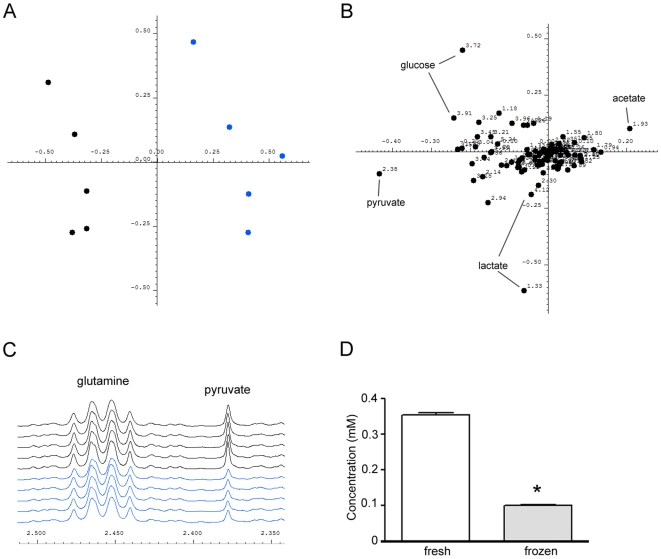
Metabonomics analysis of matched fresh (black) and frozen and thawed (blue) CMp11 samples. (**A**) Examination of the PCA score plot indicated differences between the metabolic profile of fresh CMp11 and matched samples that had undergone a freeze-thaw cycle. (**B**) By analysing the corresponding loadings plot, the main disparity between the two samples was higher levels of pyruvate in fresh CMp11. (**C**) Decreased levels of pyruvate were clearly observable in the ^1^H-NMR spectra and (**D**) accurate quantitation by NMR revealed a 44% reduction of pyruvate following the freeze-thaw cycle. *P<0.0001.

Typically, CM batches are prepared in advance and freeze stored until use. To investigate whether a freeze-thaw cycle may induce any changes in the metabolic profile of CMp11, matched samples were collected and ^1^H-NMR spectra obtained immediately or following 2 weeks storage at −20°C. Metabolic changes were analysed using PCA and as evident in the score plot (Figure 4A) differences between fresh and frozen CMp11 could be discerned. As seen in the PCA loadings plot (Figure 4B), these differences were mainly accounted for by higher concentrations of pyruvate in fresh CMp11 samples (Figure 4C). Quantification of pyruvate concentrations was performed using NOESY ^1^H-NMR spectra with these results confirming a significant decrease of pyruvate in frozen samples (Figure 4D).

**Figure 5:**
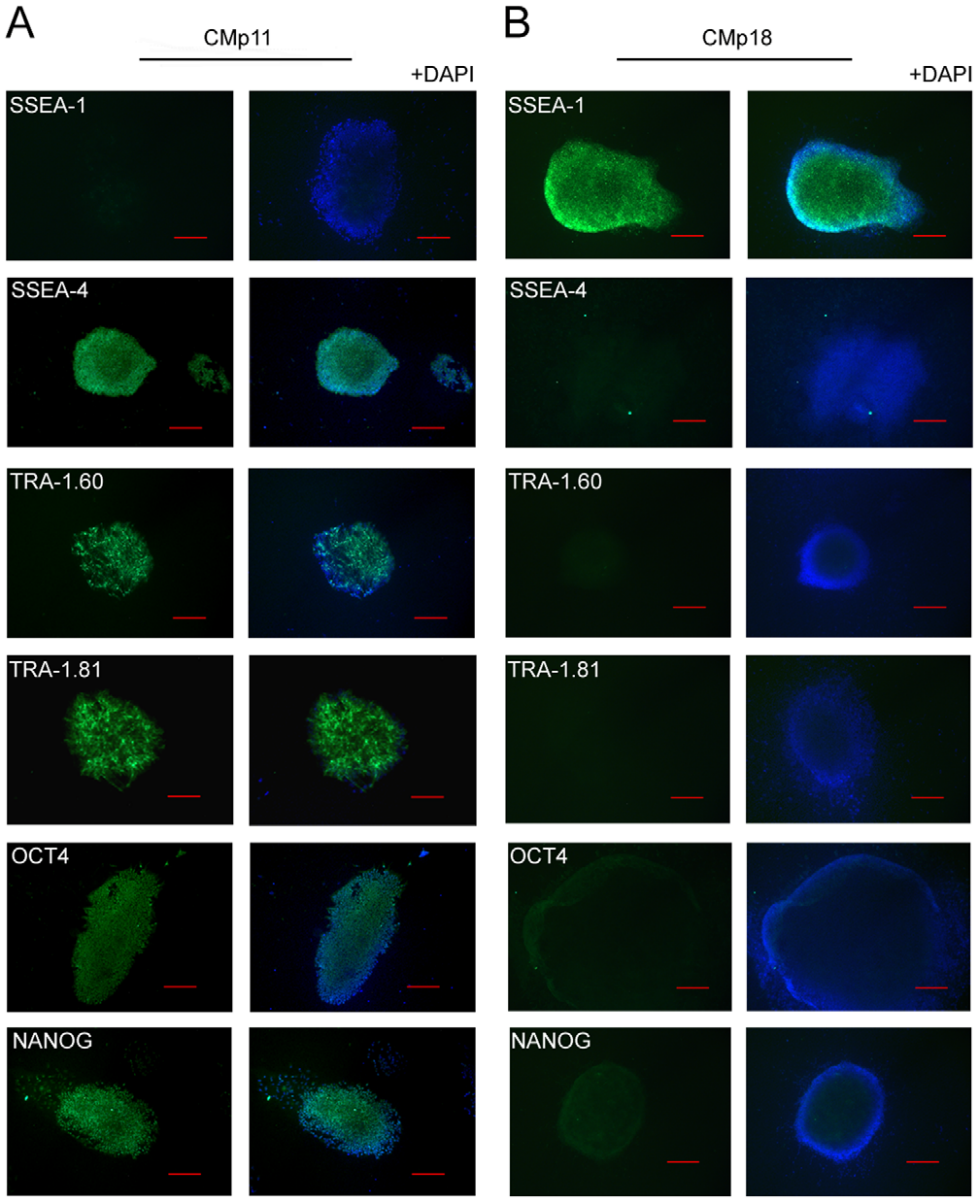
Characterisation of hESCs maintained in CMp11 and media conditioned by passage 18 HFFs (CMp18). Representative immunofluorescent images of hESCs maintained for 5 days in CMp11 (n = 5) and CMp18 media (n = 5) (bar = 100 µm, TRA-1.81 bar = 200 µm). Cells cultured in CMp11 presented well defined colonies with morphology characteristic of undifferentiated cells. In contrast, CMp18 cultured cell were morphologically distinct with colonies lacking smooth and defined edges and a clearing effect from the centre. Consistent with an undifferentiated phenotype, CMp11 cultured cells were negative for the early differentiation marker SSEA-1 (green) and positive for the undifferentiation markers SSEA-4, TRA-1.60, TRA-1.81, OCT4 and NANOG. Cells cultured in CMp18 were positive for SSEA-1 and negative for undifferentiation markers. All cells were co-labelled with the nuclear stain DAPI (blue).

**Figure 6:**
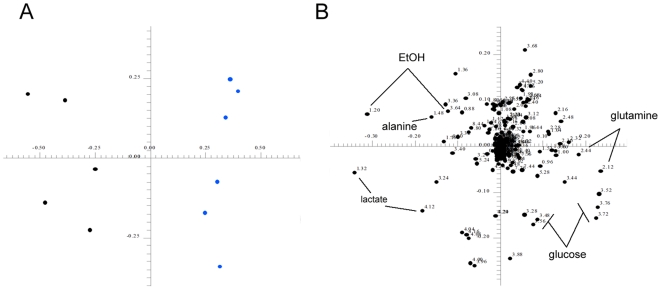
Principal Components Analysis of CMp11 and CMp18 samples. (**A**) Analysis of the metabolic profiles of CMp11 and CMp18 media by PCA revealed disparate metabolic profiles as evident in the score plot. (**B**) Examination of the corresponding loadings plot indicated CMp18 media contained characteristically higher concentrations of glucose and glutamine and comparably lower concentrations of lactate and alanine highlighting reduced metabolic activity of the passage 18 HFFs.

To indentify metabolite changes induced by conditioning that are functionally relevant to the maintenance of hESCs *in vitro*, metabolic profiling of CMp11 and media conditioned by passage 18 HFFs (CMp18) were compared (Figure 5). In CMp11, hESCs could be maintained in a pluripotent, undifferentiated state for up to 20 passages. Contrastingly, differences in the morphology of hESCs cultured in CMp18 were observed within 3 passages. hESCs cultured through 3 passages in CMp11 formed distinctive colonies with morphology consistent of undifferentiated cells while cells cultured in CMp18 were morphologically distinct producing colonies lacking smooth and defined edges. Consistent with an undifferentiated phenotype, CMp11 cultured cells were negative for the early differentiation marker SSEA-1 (green) and positive for a panel of undifferentiation markers (SSEA-4, TRA-1.60, TRA-1.81, OCT4 and NANOG). On the contrary, cells cultured in CMp18 were positive for SSEA-1 and negative for undifferentiation markers. A cassette of molecular markers of differentiation was also assessed confirming the differentiation status of both cell populations (see Figure S1). Examination of the CMp11 and CMp18 metabolic profiles by PCA revealed distinguishing features of both media (see Figure 6). Specifically, analysis of the PCA loadings plots enabled rapid identification of those metabolites with differing signal intensities and thus, disparate concentrations. CMp11 contained consistently higher concentrations of pyruvate, alanine and lactate whilst the CMp18 samples contained higher levels of glucose and glutamine.

## Discussion

Media conditioned by HFFs provides a complex mixture of protein and metabolic factors that collectively support the maintenance of pluripotent hESCs *in vitro* [11]. Recent efforts have been made to characterise the protein components of HFF conditioned media [18], yet a void of information regarding the chemical and/or metabolic component exists. Moreover, the capacity of CM to maintain pluripotent hESCs *in vitro* cannot currently be determined until days after initiating the culturing procedure. A method enabling a rapid assessment of CM functionality would likely minimise time loss and wastage of cells and reagents. In this study we have addressed these issues by characterising and quantifying the metabolic constituent of HFF conditioned media using ^1^H-NMR. This information could then be used to classify CM samples on the basis of their metabolic profiles.

To the best of our knowledge, this study represents the first application of NMR for the assessment of CM functionality for the growth of undifferentiated hESCs. Acquisition of the NMR spectra and subsequent analysis by multivariate statistics took approximately 30 min per sample mainly due to the high number of scans (256) acquired per sample. This was initially necessary to ensure a good signal to noise ratio and to detect the most ^1^H signals possible for constructing the multivariate models. However, once a multivariate model has been built and the most important spectral features identified, experimental time could be easily reduced to around 10 mins per sample further increasing high throughput possibilities.

Initial examination of the ^1^H-NMR spectra revealed a high number of proton signals originating from metabolites and small compounds present in the TeSR-1 media (conditioned by passage 11 HFFs; Figure 1). Due to the inherent quantitative nature of NMR, we were able to utilise this spectral information in two distinct ways; firstly for the classification and characterisation of samples using multivariate statistical analyses and secondly, the identification and subsequent quantification of metabolites present in the media.

As typical for metabonomics studies, ^1^H-NMR were subjected to unsupervised PCA to reduce the density of the data matrix and highlight correlated metabolic variation between samples [27]. This unbiased approach proved effective as a rapid, global assessor of metabolic differences between UM and CMp11 (see Figure 2). Alterations in the ^1^H-NMR profile induced by the conditioning process could be easily identified by interpreting corresponding PCA loadings plots and the metabolites of interest could then be identified using the Bruker NMR Metabolic Profiling Database or in-house standards. As expected of cultured human fibroblasts, CMp11 contained high concentrations of lactate [33], [34] as well as lower concentrations of the commercial L-glutamine source, GlutaMAX^TM^ that when hydrolysed by aminopeptidases in the culture media, produces detectably higher levels of alanine. Changes in the chemical shifts of HEPES were also observed indicating modifications in its chemical structure as it buffers against pH changes endured during the conditioning process.

The aforementioned changes could all be qualitatively confirmed by visual inspection of the ^1^H-NMR spectra (Figure 2C). However, to obtain a more detailed characterisation of metabolic alterations induced by HFF conditioning, quantification of 32 metabolites was undertaken. A total of 29 identified metabolites were already known to be present in the TeSR-1 media. The concentrations of 10 of these metabolites were significantly increased (representing production and/or secretion) while an additional 7 were decreased (representing utilisation) following 24 h conditioning by HFFs (See Table 1). Interestingly, low concentrations of 2-hydroxybutyric and 2-hydroxyisovaleric acids were also detected in TeSR-1 media prior to conditioning. These compounds do not appear in any published lists of TeSR-1 and their origin is currently unknown. Previous studies have identified trace excretions of 2-hydroxybutyric and 2-hydroxyisovaleric acid in media from bacterial cultures but these are typically accompanied by high lactate excretions [35]. In this study, bacterial sources of 2-hydroxybutyric acid or 2-hydroxyisovaleric acid are improbable as all TeSR-1 samples assessed were devoid of lactate and no changes in either hydroxyl acid were detected during conditioning. Despite the origin of these hydroxyl-acids, their presence in both CMp11 and UM may prove functionally relevant and furthermore, emphasises the power of ^1^H-NMR to identify previously unknown metabolites in media samples.

Three additional metabolites released into CMp11 media during conditioning were successfully identified and quantified. As previously mentioned, significant levels of lactate (4.5 mM) were observed after 24 h conditioning but µM concentrations of formate and pyroglutamic acid could also be detected. The observation of the latter in CMp11 provides a particularly good example of the utility of ^1^H-NMR for monitoring determinate reactives in culture media. As evident in Figure 2C, the GlutaMAX^TM^ dipeptide (L-alanyl-L-glutamine) present in UM is hydrolysed by aminopeptidases during the conditioning process forming alanine and glutamine. However, glutamine concentrations did not increase during conditioning most likely because of HFF utilisation [33] and previously described spontaneous, non-enzymatic decomposition of glutamine to pyroglutamic acid in culture conditions [36]. Collectively, the aforementioned results clearly demonstrate that the chemical environment hESCs are exposed to during culture and maintenance in CMp11 differs substantially to that of UM. While further experiments are required to examine the functional importance of each of these changes, this information can be utilised for future design of “ready to use” chemically defined media for maintenance of hESCs *in vitro*.

Analysis of CMp11 samples collected during a time course of 72 h revealed marked differences in the metabolic profiles (Figure 3). Decreased formation of lactate and pyruvate as well as decreased utilisation of glucose and glutamine in samples of CMp11 collected from 48 h and 72 h indicate slowing growth rates and metabolic activity of these cells. The presence of increased EtOH concentrations in samples collected after 72 h was surprising, however, its metabolic breakdown by HFFs likely accounts for the accompanying high levels of acetate. Testing for the presence of microbial organisms capable of producing and excreting EtOH by fermentation proved negative and we did not detect any other products typical of non-mammalian metabolic pathways. Although EtOH is present in TeSR-1 media as a solvent, increased levels are most likely due to accidental introduction during sterilisation procedures undertaken with media changes as described by others [32]. It is important to note that these levels of EtOH did not appear to affect the growth rate of the hESCs. These results highlight the utility of monitoring metabolic components of CMp11 to ensure reproducibility between sample batches. Moreover, these results suggest that the common practice of pooling samples of conditioned media prepared over various days may mask functionally critical differences between media batches.

Previous metabolomics studies on blood samples and other body fluids have found that storage of samples at −20°C is not sufficient for suspending metabolic activity [37], [38]. We have previously observed that fresh samples of CMp11 that successfully maintain hESCs *in vitro* in an undifferentiated state lose their capacity to do so after long periods of freeze storage. Comparison of the metabolic profile of paired fresh and frozen CMp11 samples indicated that a 2 week freeze-thaw cycle at −20°C is sufficient to induce changes demonstrable by ^1^H-NMR (Figure 4). Increased acetate coupled with decreased pyruvate point toward pyruvate decarboxylation via the pyruvate dehydrogenase complex during the freezing or thawing process. In any case, the results are indicative of ongoing enzymatic activity during storage of CMp11 and suggest snap freezing and subsequent storage at −80°C, or the use of fresh media, would likely minimise variance between media preparations.

In our hands, hESCs cultured in CMp18 spontaneously differentiate before 5 days in culture, confirmed by their increased expression of numerous differentiation markers and the loss of expression of the early undifferentiation marker SSEA-1 (Figure 5). Metabolic profiling of CMp18 samples revealed consistently lower lactate and alanine concentrations than CMp11 as well as higher glucose and glutamine concentrations indicating lower metabolic activity of passage 18 HFFs. Interestingly, this reflects the metabolic profile of passage 11 HFFs cultures for 72 h (Figure 3) suggesting both cell age and passaging act detrimentally on metabolic activity. It is likely that the observed metabolic changes reflect important functional protein changes that may additionally play an importantly role in the maintenance of hESCs *in vitro*. This experiment highlights the capacity of the ^1^H-NMR-based metabolomics to rapidly classify similar, yet functionally disparate CM samples. We are now undertaking further experiments to explore and functionally characterise the mechanisms responsible for disparate metabolite production observed in the passage 18 HFFs. Nevertheless, we envisage that a prompt assessment of CM by ^1^H-NMR would prevent the loss of valuable experimental time, cells and reagents caused by having to culture cells over a number of days before learning the suitability of the prior conditioning procedure.

In conclusion, our results show that ^1^H-NMR based metabonomics is a useful and rapid tool for classifying and identifying media batches that may functionally differ due to variance encountered during the conditioning process or even during storage. The characterisation and quantification of the metabolic component of CM by ^1^H-NMR offers new insight on the precise chemical constituents of functionally effective media and may facilitate future design of media recipes for hESC growth or their differentiation products.

## Materials and Methods

Unless otherwise stated, materials and reagents were purchased from GIBCO, Invitrogen (Carlsbad, CA). All experimental procedures were approved by the ethics and research committees of the Centro de Investigación Príncipe Felipe, Valencia, Spain.

### Preparation of human fibroblast conditioned media

Commercially available human foreskin fibroblasts (HFFs- American Type Culture Collection No. CRL-2429, Passage 11) were cultured in Iscovés Modified Dulbeccós Medium (IMDM) (Sigma, St. Louis, MO) supplemented with 10% heat-inactivated fetal bovine serum (Lonza, Basel, Switzerland), 2% GlutaMAX^TM^ and 1% Penicillin/Streptomycin. Cells were split using TrypLE Select every 5–7 days. When cells reached 80–90% confluence they were mitotically inactivated using 10 µg/ml mitomycin C (Sigma, St. Louis, MO) for 2.5 h. Following three washes with PBS, cells were seeded (7.5×10^6^ cells) in 0.1% gelatin coated T75 flasks. After 24 h the media was replaced with TeSR-1 media [39]. The conditioned TeSR-1 media (CM) was collected every 24 h until the 5^th^ day before being pooled and stored at −20°C. To investigate any alterations in the metabolic profile that may be induced by a freeze-thaw cycle, ^1^H-NMR spectra were acquired from both fresh CM samples and matched frozen samples at −20°C for 2 weeks. To examine the effect of cell passaging on the media preparation, CM was also collected as described using passage 18 HFFs.

### hESC culture on ECM with Conditioned Media

Primary hESCs (H9 line, WiCell Inc., Madison, WI) were cultured and maintained on ECM coated plates as we have previously reported [40], [41]. hESC were mechanically dissociated and passaged onto ECM coated plates with CM prepared from passage 11 (CMp11) or passage 18 HFFs (CMp18). Cells were passaged to new coated plates every 5–7 days and maintained at 5% CO_2_ and 37°C during at least 4 passages to ensure maintained undifferentiation and pluripotency.

### Characterization of hESCs

The efficiency of CM to maintain hESCs in an undifferentiated state was characterised by both molecular and protein-based methods as we have previously reported [40]. Briefly, hESCs were characterized by immunohistochemistry using antibodies against a cassette of undifferentiated hESCs markers including SSEA4, TRA-1-60, TRA-1-81, OCT-4 (Millipore, Billerica, MA) and NANOG (R&D systems, Minneapolis, MN). Additionally, expression of the differentiation marker SSEA1 (Millipore) was also examined. Firstly, hESCs were fixed in 4% paraformaldehyde (Electron Microscopy Sciences, Hatfield, PA) for 15 min at room temperature before permeabilising them in 0.1% Triton-X-100 (Sigma) diluted in PBS for 30 min. Samples were then blocked in blocking solution (10% goat serum + 1% BSA for all except NANOG; 10% donkey serum + 1% BSA) for 45 min at room temperature before being incubated for 60 min at room temperature with primary antibodies (1∶100 dilution in blocking solution for all except NANOG- 1∶20 dilution). Following three 5 min rinses in PBS, samples were incubated with the appropriate Alexa-Flour 488 secondary antibodies (1∶500 dilution, Sigma, St. Louis, MO) for 60 min at room temperature in darkness. Finally, samples were washed 3 times with PBS for 5 min and mounted using Prolong Gold Antifade Reagent containing DAPI (Invitrogen) for nuclear staining. Images were acquired using a Zeiss Axiovert 200 M fluorescence microscope (Zeiss, Oberkochen, Germany).

### CM sample preparation and NMR Acquisition

A total of 495 µl CM was added to a 5 mm NMR tube along with 55 µl D_2_O containing 1 mM 3-(trimethylsilyl)propionic - 2, 2, 3, 3 - D4 acid sodium salt (TSP; Eurisotop, Gif-Sur-Yvette, France), as an internal reference for calibration and quantification. NMR acquisition was performed immediately using a Bruker Avance II 600 spectrometer (Bruker Biospin, Rheinstetten, Germany) equipped with a TCI cryoprobe operating at 600.13 MHz for ^1^H. To facilitate the examination of low molecular weight metabolites, relaxation edited ^1^H-NMR spectra were acquired at 300 K using a Carr-Purcell-Meiboom-Gill (CPMG) pulse sequence [42] with a total of 256 free induction decays (FIDs) collected into 64K data points over a spectral width of 20 ppm. A 3 s relaxation delay was applied between FIDs. A continual water presaturation RF field of 35 Hz was also used with the total spin-spin relaxation delay being 40 ms. It is known that the signal intensity derived by CPMG can be influenced by the relaxation properties of a given chemical group. Importantly, any possible reduction in signal intensity caused by relaxation effects on a signal would be consistent across samples; thus, relative changes in concentration values would be still precise and biologically relevant. However, for more accurately quantifying target metabolites, 1D-nuclear Overhauser effect spectroscopy (NOESY) spectra were collected using a 4 s relaxation delay, 10 ms of mixing time and 128 FIDs. The first transient of the 2D experiment was acquired in order to obtain good water suppression. The pulse sequence used incorporates gradients so a shorter mixing time can be used in turn avoiding relaxation issues during the mixing and decreasing the error introduced in the quantification. The sequence incorporated a pre-saturation pulse during the relaxation time in order to decrease the water signal intensity. For both experiments, an exponential function equivalent to that of a 1.0 Hz line-broadening factor was applied to the FIDs followed by Fourier transformation. Spectral phasing and baseline correction procedures were performed automatically using TopSpin (v 2.1, Bruker BioSpin, Rheinstetten, Germany). Spectra were also visually inspected and adjusted manually if necessary. Standard protocols are described further by Beckonert *et al.* [43]

### Spectral Data Processing and Preparation for Statistical Analysis

Calibration of spectra was performed to TSP at chemical shift (δ) = 0.00 ppm. Metabolite assignments were achieved using Amix (v 3.8.3, Bruker BioSpin, Rheinstetten, Germany) in combination with the Bruker NMR Metabolic Profiling Database (BBIOREFCODE 2.0.0, Bruker BioSpin, Rheinstetten, Germany) along with in-house standards. To facilitate metabolite identification we also acquired 2D experiments including *J*-resolved ^1^H (*J*-res) and 2D ^1^H,^13^C-HSQC using previously reported settings [44]. To convert the ^1^H-NMR spectra into a format conducive to statistical analysis, the region of the NMR spectra containing metabolite resonances (0.1–10.0 ppm) was divided into buckets of 0.04 ppm and integrated. The region of spectra corresponding to residual water (4.5–5.5 ppm) was excluded from the analysis. In the case of quantitation of target metabolites, each region of the NOESY spectra corresponding to a metabolite signal was selected and integrated in Amix using the Variable Sized Bucketing module and concentrations calculated with respect to the 1 mM TSP internal standard. This method of bucketing was also used to compare differences in metabolite signals between time course collections of CM and frozen and thawed samples. Purified metabolite ^1^H-NMR profiles in the BBIOREFCODE (Bruker Biospin) were used to help determine variable sized regions for integration. If possible, both singlets and multiplets of the same metabolite were used for integration and quantification. Buckets were normalised to the sum of total spectral intensity to minimise possible differences in concentration between samples and to reduce any residual minor chemical shift alterations [45], [46].

### Statistical Analysis

To examine any inherent underlying variation between the metabolic profiles of the media samples, unsupervised Principal Components Analysis (PCA) was employed (Amix v 3.8.3). For this, data was Pareto scaled whereby the square root of the standard deviation was used as the scaling factor [47]. This method is commonly employed in metabonomics as a way to decrease the relative importance of highly abundant metabolites in the data set whilst maintaining data structure. The first two principal components plotted and examined for any clustering trends. Corresponding loadings plots were used to identify metabolites responsible for any contribution to separation observed in the PCA score plots.

For univariate statistical analyses, results were presented as mean ± standard error of the mean. Statistical significance was determined using Student's t-test or ANOVA (Tukey-Kramer *post hoc* test) where appropriate. Significance was assumed where *P*<0.05.

## Supporting Information

Figure S1 **Molecular characterisation of hESCs maintained for 4 passages in CMp11 or CMp18.** Real Time RT-PCR was performed on a cassette of differentiation markers to confirm the molecular status of hESCs in culture. Gene expression levels of the ectoderm marker - paired box gene 6 (PAX6), the mesoderm marker- brachyury (BRA) and the endoderm marker- α-fetoprotein (AFP) were normalised to GAPDH and then compared to levels present in undifferentiated H9 passage 53 cells (H9p53). The results indicate that colonies cultured in CMp18 batches over 4 passages predominately differentiate toward ectoderm and to a lesser extent, mesoderm. No detectable differentiation toward endoderm was observed as indicated by expression levels of AFP lower than that observed in H9p53 cells. The data are presented as mean ± SD (n = 5).
(DOC)

Table S1 **Comparison of calculated metabolite concentrations media and ^1^H-NMR determined values in TeSR-1 media.** Concentrations of 20 metabolites present in TeSR-1 media were calculated using published recipes for media components and compared with values determined experimentally by ^1^H-NMR. Values were generally highly consistent indicating quantitative accuracy of ^1^H-NMR, however in some cases, metabolite concentrations where overestimated due to overlapping signals (*) or underestimated due to the close proximity of the water signal (**) and subsequent altering of the baseline. Lowest and highest metabolite concentrations differed by almost four orders of magnitude.
(DOC)
