# Supplementary figures and images for: Characterisation of Human Embryonic Stem Cells Conditioning Media by 1H-Nuclear Magnetic Resonance Spectroscopy
Source: PLoS One. 2011 Feb 9;6(2):e16732. doi: 10.1371/journal.pone.0016732 (PMC3036660; doi:10.1371/journal.pone.0016732)

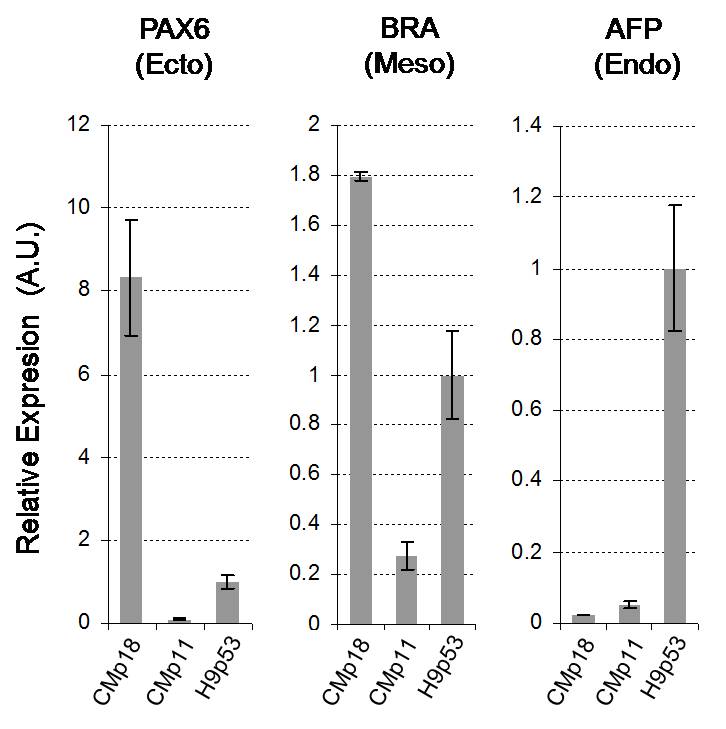

Supplement: Figure S1 — Molecular characterisation of hESCs maintained for 4 passages in CMp11 or CMp18. Real Time RT-PCR was performed on a cassette of differentiation markers to confirm the molecular status of hESCs in culture. Gene expression levels of the ectoderm marker - paired box gene 6 (PAX6), the mesoderm marker- brachyury (BRA) and the endoderm marker- α-fetoprotein (AFP) were normalised to GAPDH and then compared to levels present in undifferentiated H9 passage 53 cells (H9p53). The results indicate that colonies cultured in CMp18 batches over 4 passages predominately differentiate toward ectoderm and to a lesser extent, mesoderm. No detectable differentiation toward endoderm was observed as indicated by expression levels of AFP lower than that observed in H9p53 cells. The data are presented as mean ± SD (n = 5). (DOC) [file pone.0016732.s001.doc]
