# Supplementary material for: Characterisation of Human Embryonic Stem Cells Conditioning Media by 1H-Nuclear Magnetic Resonance Spectroscopy
Source: PLoS One. 2011 Feb 9;6(2):e16732. doi: 10.1371/journal.pone.0016732 (PMC3036660; doi:10.1371/journal.pone.0016732)
Supplement: Table S1 — Comparison of calculated metabolite concentrations media and 1H-NMR determined values in TeSR-1 media. Concentrations of 20 metabolites present in TeSR-1 media were calculated using published recipes for media components and compared with values determined experimentally by 1H-NMR. Values were generally highly consistent indicating quantitative accuracy of 1H-NMR, however in some cases, metabolite concentrations where overestimated due to overlapping signals (*) or underestimated due to the close proximity of the water signal (**) and subsequent altering of the baseline. Lowest and highest metabolite concentrations differed by almost four orders of magnitude. (DOC) [file pone.0016732.s002.doc]

| **Metabolite** | **Calculated** | **Experimental** | **Chemical Shift (ppm)** | **Multiplicity** | **Assignment** |
| --- | --- | --- | --- | --- | --- |
| glutamate | 0.139 | 0.556±0.028 | 2.08 | m | half β-CH_2_ |
| glutamax | 1.000 | 1.209±0.050 | 1.52 | d | CH_3_ |
| glutamine | 1.963 | 2.065±0.096 | 2.45 | m | half γ-CH_2_ |
| histidine | 0.118 | 0.118±0.004 | 7.07 | s | H4 |
| isoleucine | 0.327 | 0.355±0.016 | 1 | d | β-CH_3_ |
| leucine | 0.354 | 0.369±0.016 | 0.97 | d+d | -δ-CH_3_ |
| methionine | 0.091 | 0.132±0.008 | 2.65 | t | -γ-CH_2_ |
| phenylalanine | 0.169 | 0.196±0.009 | 7.39 | m | H2,3,4,5+6 |
| tryptophan | 0.035 | 0.036±0.001 | 7.75 | d | CH |
| tyrosine | 0.168 | 0.197±0.009 | 7.2 | d | CH |
| valine | 0.355 | 0.361±0.014 | 1.03 | d | CH_3_ |
| choline | 0.050 | 0.065±0.003 | 3.21 | s | N(CH_3_)_3_ |
| folate | 0.005 | 0.001±0.000 | 6.42 | d | CH |
| i-inositol | 0.055 | 0.035±0.003 | 4.07 | s | H5 |
| niacinamide | 0.013 | 0.010±0.000 | 8.94 | s | NCH |
| pantothenic acid | 0.004 | 0.014±0.001 | 0.89 | s | CH_3_ |
| pyridoxine | 0.008 | 0.013±0.001 | 7.66 | s | H6 |
| thiamine | 0.005 | 0.012±0.001 | 9.42 | s | CH |
| glucose | 13.854 | 15.826±0.688 | 5.24 + 3.9 | d + dd | H1 + half CH2-C6 |
| HEPES | 11.791 | 13.254±0.561 | 3.12-3.18 | m | CH_2_(SO_3_) |

Abbreviations: s-singlet; d-doublet; dd-double doublet, t-triplet; q-quartet; m-multiplet. Chemical shifts referenced to TSP at 0.00 ppm. Values reported as mean values (n = 5) ± standard deviation (SD).
